# Odor Removal Characteristics of a Laminated Film-Electrode Packed-Bed Nonthermal Plasma Reactor

**DOI:** 10.3390/s110605529

**Published:** 2011-05-25

**Authors:** Takuya Kuwahara, Masaaki Okubo, Tomoyuki Kuroki, Hideya Kametaka, Toshiaki Yamamoto

**Affiliations:** 1 Department of Mechanical Engineering, Osaka Prefecture University, 1-1 Gakuen-cho, Naka-ku, Sakai 599-8531, Japan; E-Mails: takuya.kuwahara@gmail.com (T.K.); kuroki@me.osakafu-u.ac.jp (T.K.); 2 Engineering and Technology Center, Niigata Power Systems Co., Ltd., 125-1 Nishishin-machi, Ota-shi, Gunma 373-0847, Japan; E-Mail: hkame1@hotmail.com; 3 Department of Electrical and Electronic Engineering, Tokyo City University, 1-28-1 Tamazutsumi, Setagaya-ku, Tokyo 158-8557, Japan; E-Mail: yama-t@tcu.ac.jp

**Keywords:** plasma, chemical reaction, nonthermal plasma, odor control, ammonia, acetaldehyde, living environment, packed-bed

## Abstract

Odor control has gained importance for ensuring a comfortable living environment. In this paper, the authors report the experimental results of a study on the detailed characteristics of a laminated film-electrode and a laminated film-electrode packed-bed nonthermal plasma reactor, which are types of dielectric barrier discharge (DBD) reactor used for odor control. These plasma reactors can be potentially used for the decomposition of volatile organic compounds (VOCs) and reduction of NO_x_. The reactor is driven by a low-cost 60-Hz neon transformer. Removal efficiencies under various experimental conditions are studied. The complete decomposition of the main odor component, namely, NH_3_, is achieved in a dry environment. The retention times are investigated for the complete removal of NH_3_ in the case of the film-electrode plasma reactor and the film-electrode packed-bed plasma reactor. The removal efficiency of the former reactor is lower than that of the latter reactor. Mixing another odor component such as CH_3_CHO in the gas stream has no significant effect on NH_3_ removal efficiency.

## 1. Introduction

Odor control has gained importance for ensuring a comfortable living environment. Previously, the authors and other researchers have reported the implementation of a nonthermal plasma odor control system in an animal house [1,2], odor removal from restaurants, and cigarette smoke removal [3–6]. A nonthermal plasma is typically referred to as an atmospheric-pressure nonequilibrium plasma, in which the gas temperature is considerably lower than the electron temperature. For this type of plasma, the gas temperature is virtually equal to the atmospheric temperature, and the electron temperature is of the order of 1–10 eV or several tens of thousands of K. Nonthermal plasmas can enhance chemical reactions with low applied electrical energy.

Recently, large odor-removal aftertreatment systems using nanosecond pulsed corona plasma combined with adsorbents or catalysts were developed for public garbage incineration plants [7], compost factories [8,9] and NO_x_ removal [10]. However, the capital cost involved in the development of these systems is very high because an extremely fast pulse-switching (rising time, ∼100 ns) high-voltage power supply is required for high performance. For small apparatus such as a commercial garbage processor [11], odor control is usually performed by using biochip technology combined with oxidation catalysts. However, this process is extremely slow and not so effective. Therefore, low-cost, smaller, faster and more effective odor removal systems are required. In order to meet this requirement, the authors have investigated the use of a packed-bed plasma system for odor removal. This plasma reactor uses packed pellets with a high relative permittivity (∼10,000) [12].

In this paper, the authors report the experimental results of a study on the detailed characteristics of laminated film-electrode and laminated film-electrode packed-bed plasma reactors, which are driven by a low-cost 60-Hz neon transformer and can be combined with an indoor air cleaner, used for the removal of ammonia (NH_3_), a typical odor component. The effects of an initial concentration and a flow rate on removal efficiencies are studied. Further, the decomposition tendency of a mixture of NH_3_ and acetaldehyde (CH_3_CHO) is studied. Byproducts in the odor removal process are discussed.

## 2. Experimental Apparatus and Method

### 2.1. Experimental Setup

**Figure 1.**
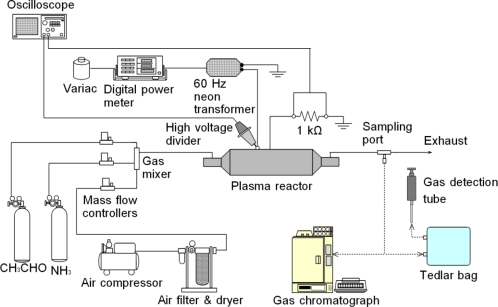
Schematic diagram of experimental setup for NH_3_ and CH_3_CHO decomposition.

The experimental setup is shown in Figure 1. Ammonia (950 ppm) balanced with N_2_ and acetaldehyde (1020 ppm) balanced with N_2_ prepared in cylinders and dry air supplied through a dryer (relative humidity is approximately 4%) by an air compressor are mixed using mass flow controllers and a gas mixer. The regulated gas is passed through the plasma reactor. The gas flow rates are set to 5, 10 and 15 L/min. After the flowing gas and plasma achieve steady state conditions, the treated gas is sampled in a Tedlar bag through a sampling port. The concentration of NH_3_ is measured at the Tedlar bag using a gas detection tube (Gastec GV-100S) whose minimum scale value is 2.5 ppm. It is noted that the measurement with the gas detection tube includes an inaccuracy of 1 ppm for a measured value less than 20 ppm and an inaccuracy of 2 ppm for a measured value for 20–100 ppm. In the treatment of the NH_3_ and CH_3_CHO mixture, a 50 mL gas sample is collected from the sampling port using a glass syringe. The efficiency of CH_3_CHO removal from the sample is evaluated using a flame ionization detector (FID) gas chromatograph (Shimadzu GC-14B).

### 2.2. Plasma Reactors

**Figure 2.**
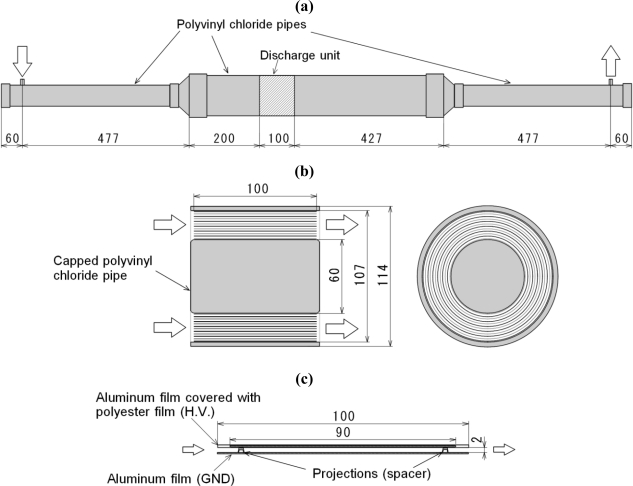
Laminated film-electrode plasma reactor. **(a)** Schematic of odor removal test section; **(b)** Configuration of plasma reactor (Discharge unit); **(c)** Detail of laminated film electrodes.

The laminated film-electrode plasma reactor shown in Figure 2, which is a kind of DBD reactor, is used for the removal of gaseous NH_3_ and CH_3_CHO. This reactor can treat higher flow rate gas than the ordinary glass tube-type plasma reactor [12]. Figure 2(a) shows the schematic diagram of the plasma reactor, Figure 2(b) shows the frontal and cross sectional views of the discharge unit and Figure 2(c) shows the details of the electrodes. The plasma reactor comprises two types of film-electrodes. One has projections of each 2 mm in size and it is used as a ground electrode as shown in Figure 2(b). The other is covered by a polyester film which is used for dielectric barrier and it is used as a discharge electrode. These films have a width of 100 mm. Nonequilibrium nonthermal plasma is generated between two films and the distance between them is 2 mm. As shown in Figure 2(b), the two films are wrapped around a capped polyvinyl chloride pipe of 60 mm diameter with alternating layers. The laminated film-electrode packed-bed reactor is a film-type reactor whose electrodes are spaced by 2 mm. It is packed with BaTiO_3_ pellets (diameter, 1.0 mm; relative dielectric constant at room temperature, 10,000). In this case, nonthermal plasma is induced among the pellets. It would be possible to achieve the odor removal with other kind of pellets. Although some tests with other kinds of pellets should be required as future works, the odor removal tests only with BaTiO_3_ are carried out in the present study.

A high AC voltage (max. *V*_p_ = 20 kV and 20 mA) of 60 Hz is applied to the reactor using a 60-Hz neon transformer. Although there are other choices for the type of power supply such as pulsed voltage, the AC voltage has an advantage from the point of view of cost. The input power of the neon transformer is measured using a digital power meter (Yokogawa WT 110 E). The applied voltage waveform is measured using an oscilloscope (Tektronix TDS380P-2GS/s) through a high voltage divider. The current waveform is obtained by measuring the voltage to 1 kΩ resistance which is connected in series between the plasma reactor and the ground. The discharge power is calculated from multiplying the voltage and current waveforms. In the results, the voltage applied to the reactor is represented in the form of the peak-to-peak voltage (*V*_p-p_) on the basis of the ground.

**Figure 3.**
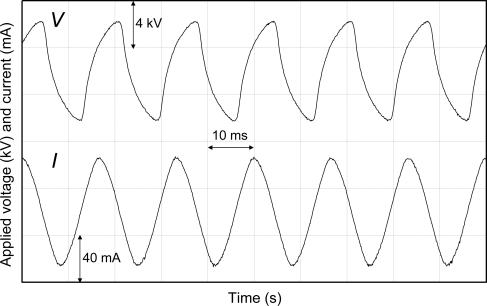
Applied voltage and electric current in laminated film-electrode plasma reactor.

**Figure 4.**
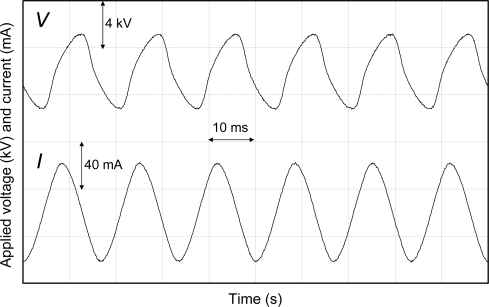
Applied voltage and electric current in laminated film-electrode packed-bed plasma reactor.

Figures 3 and 4 show the representative waveforms of applied voltage and electric current of the film-electrode with the applied voltage of 8.5 kV and film-electrode packed-bed plasma reactors with the applied voltage of 6.5 kV, respectively.

**Figure 5.**
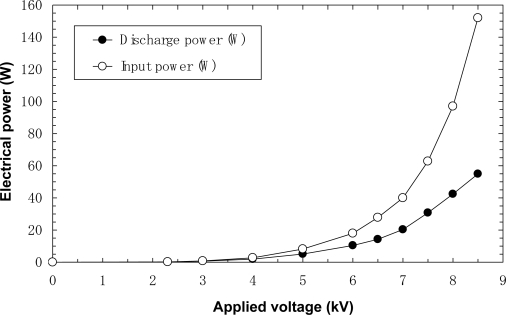
Electrical power *vs.* applied voltage for laminated film-electrode plasma reactor.

**Figure 6.**
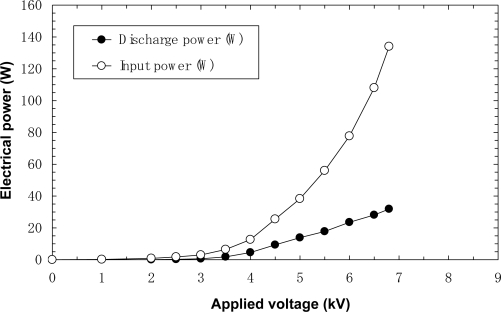
Electrical power *vs.* applied voltage for laminated film-electrode packed-bed plasma reactor.

Figures 5 and 6 show the input powers and discharge powers of the film-electrode and film-electrode packed-bed plasma reactors, respectively. It is seen from these graphs that more than 24% of the input electrical power is converted into discharge plasma power at the maximum voltage.

## 3. Results and Discussion

### 3.1. Effects of Initial Concentration, Flow Rates on NH_3_ Removal Efficiencies (Laminated Film-Electrode Plasma Reactor)

**Figure 7.**
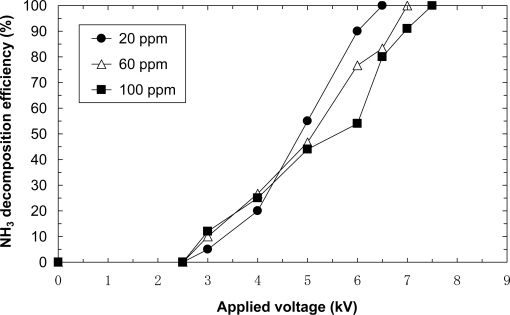
Effect of initial concentration on ammonia decomposition (flow rate = 5.0 L/min, laminated film-electrode plasma reactor).

Figure 7 shows the removal efficiencies of 20, 60 and 100 ppm NH_3_ balanced with dry air at a flow rate of 5.0 L/min. This figure reveals that the removal occurs at an applied voltage of 2.5 kV. For each initial concentration 100% NH_3_ decomposition efficiency is achieved. The resulting voltages for 20, 60 and 100 ppm are 6.5, 7.0 and 7.5 kV, respectively. Further, the result shows that a higher applied voltage is required for complete decomposition at a higher initial concentration. It is observed that the relationship is the opposite at low applied voltage under 5 kV. In general, chemical reactions with nonthermal plasma are enhanced by high concentrations of target components. The present reaction is also proportional to the applied voltage. Therefore, it is possible that the decomposition efficiency of lower initial concentrations such as 20 ppm becomes smaller at low applied voltage. The decomposition efficiency of lower initial concentrations reaches fast to 100% since the amount of NH_3_ is a little. Therefore, the decomposition efficiency of lower initial concentrations should be higher at high applied voltage. The opposite relationship at lower applied voltage can also be caused by the measurement errors of 1, 2 and 5% for 100 ppm, 60 ppm and 20 ppm cases, respectively. These are reasons why the relationship is opposite at low applied voltage.

**Figure 8.**
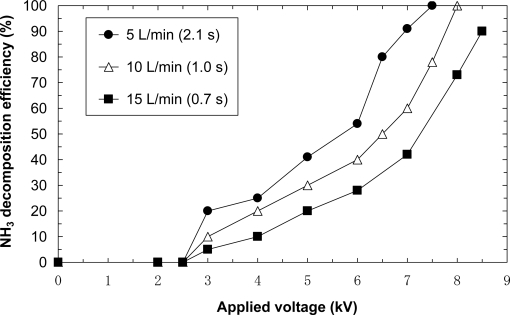
Effect of flow rate ammonia decomposition (initial concentration = 100 ppm, laminated film-electrode plasma reactor).

Figure 8 shows the removal efficiencies due to NH_3_ balanced with dry air at flow rates of 5, 10 and 15 L/min (retention times of 2.1 s, 1.0 s and 0.7 s, respectively). The initial concentration is 100 ppm. As seen in the figure, 100% NH_3_ decomposition efficiency is achieved at flow rates of 5 and 10 L/min. The applied voltages result to be 7.5 and 8.0 kV for flow rates of 5 and 10 L/min. However, 100% decomposition is not achieved at a flow rate of 15 L/min. At a flow rate of 15 L/min, a voltage higher than 8.5 kV cannot be applied because of the limitations of the transformer. It is possible to achieve complete decomposition by applying a higher voltage at a flow rate of 15 L/min. Several experiments are carried out under the same conditions. The resulting data showed little difference.

### 3.2. Effects of Initial Concentration, Flow Rate on NH_3_ Removal Efficiencies (Laminated Film-Electrode Packed-Bed Plasma Reactor)

**Figure 9.**
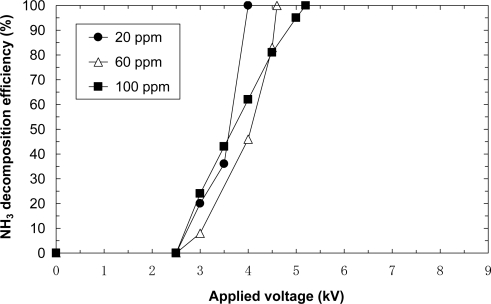
Effect of initial concentration on ammonia decomposition (flow rate = 5.0 L/min, laminated film-type packed-bed plasma reactor).

Figure 9 shows the removal efficiencies of 20, 60 and 100 ppm NH_3_ balanced with dry air at a flow rate of 5.0 L/min. This figure reveals that the removal occurs at an applied voltage of 2.5 kV. For each initial concentration 100% NH_3_ decomposition efficiency is achieved. The resulting voltages for 20, 60 and 100 ppm are 4.0, 4.6 and 5.2 kV, respectively, which are lower than voltages of the laminated film-electrode plasma reactor. As a result, the similar characteristics to the laminated film-electrode plasma reactor are obtained. As compared to the laminated film-electrode plasma reactor, the laminated film-electrode packed-bed plasma reactor achieves higher decomposition efficiency at a lower applied voltage. In other words, the film-electrode packed-bed plasma reactor consumes less power to decompose NH_3_. Similarly to Figure 7, it is also observed that the relationship is opposite at low applied voltage under 4 kV. It is probably due to the same reasons aforementioned in Figure 7.

**Figure 10.**
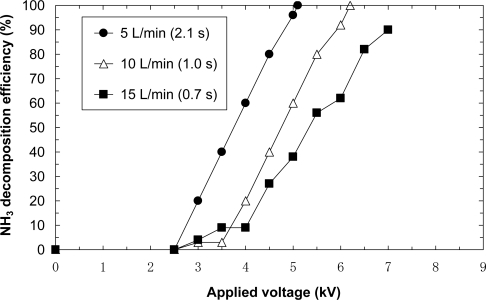
Effect of flow rate on ammonia decomposition (initial concentration = 100 ppm, laminated film-electrode packed-bed plasma reactor).

Figure 10 shows the removal efficiencies due to NH_3_ balanced with dry air at flow rates of 5, 10 and 15 L/min (retention time of 2.1 s, 1.0 s and 0.7 s, respectively). The initial concentration is 100 ppm. As seen in the figure, 100% NH_3_ decomposition efficiency is achieved at flow rates of 5 and 10 L/min and the applied voltages are 5.1 kV and 6.2 kV for the flow rates of 5 and 10 L/min, respectively. These voltages are lower than those of the laminated film-electrode plasma reactor. However, 100% decomposition is not achieved at a flow rate of 15 L/min. At a flow rate of 15 L/min, a voltage higher than 7 kV cannot be applied because of the limitation of the transformer.

As for the other kinds of plasma reactor for NH_3_ removal, a report [13] describes 20% removal efficiency for an initial concentration of 15 ppm and another report [14] mentions around 80% removal efficiency for initial concentrations of 75 and 150 ppm. In comparison with these methods, the present method achieves 100% decomposition efficiency for NH_3_ removal.

### 3.3. Effect of CH_3_CHO Mixing on NH_3_ Removal

**Figure 11.**
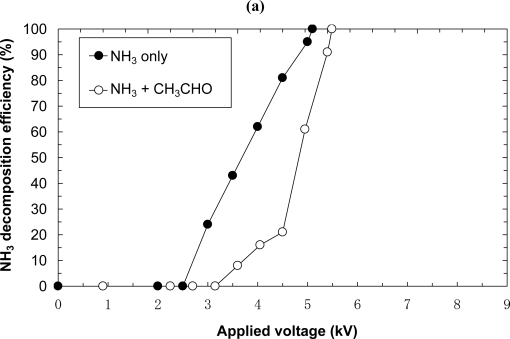

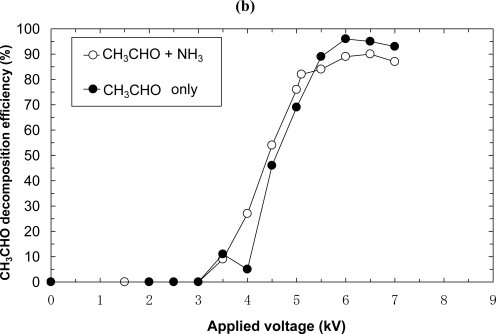
Effects of mixture of acetaldehyde and ammonia (Flow rate = 5.0 L/min, initial concentration = 100 ppm, laminated film-electrode packed-bed plasma reactor). **(a)** NH3 decomposition; **(b)** CH3CHO decomposition.

In a previous paper [15], one of the authors reported the experimental results for the decomposition of a benzene and toluene mixture using a nonthermal plasma. Thus, it was found that the component with the higher removal efficiency in a single treatment is removed with higher efficiency, and the component with the lower removal efficiency in a single treatment is removed with lower efficiency. A comparison between the decomposition efficiencies of a single gas and a mixed gas is of particular interest. Thus, a decomposition experiment for the mixture of NH_3_ and CH_3_CHO, a representative foul odor, is performed using the laminated film-electrode packed-bed plasma reactor. The mixture is prepared by mixing 100 ppm of NH_3_ and 100 ppm of CH_3_CHO. The experiment is carried out at a flow rate of 5 L/min by regulating the applied voltage. Figure 11 shows the decomposition results of NH_3_ and CH_3_CHO. Figure 11(a) shows that NH_3_ in the mixed gas is completely decomposed. The decomposition tendency is similar to that of NH_3_ as a single gas. Figure 11(b) shows that the decomposition efficiencies of CH_3_CHO in a mixed gas and CH_3_CHO as a single gas are enhanced by applying a higher voltage. However, the efficiencies are decreased at around 6 kV; thus, complete decomposition is not achieved. The reasons for the decreased efficiency around 6 kV can be the fact that the efficiency of a chemical reaction with nonthemal plasma is proportional to the concentration of target component. The concentration of CH_3_CHO decreases along with higher applied voltage due to the progress of the CH_3_CHO decomposition reactions [16], hence, the decomposition efficiencies are decreased at high voltage, *i.e.*, around 6 kV.

The thermal loss at high voltage also influences the decreases of the decomposition efficiencies. In the case of NH_3_, it appears that HNO_3_ which is generated as a byproduct contributes to the NH_3_ removal [3]. Although the concentration of NH_3_ is decreased by the reaction, HNO_3_ is generated as shown hereinafter in the reaction (13). Because this generated HNO_3_ also enhances the NH_3_ decomposition as shown hereinafter in the reaction (14), the decomposition efficiencies are maintained at high voltage. The results indicate that there is an optimal applied voltage for both the mixed gas and CH_3_CHO as a single gas. In the comparison with the other kinds of plasma reactor for CH_3_CHO removal [5,6], although the accurate comparison is difficult because the gas concentrations are different, the plasma reactor in the present paper provides the higher decomposition efficiency.

### 3.4. Comparison between Performances of Laminated Film-Electrode and Laminated Film-Electrode Packed-Bed Plasma Reactors

The plasma reactors are evaluated by using the specific energy (*SE*). *SE* is defined by:

(1) SE = W/Q (W min/L)

where *W* is the discharge power (W) and *Q* is the flow rate (L/min). Table 1 shows the resulting *SE* of complete decomposition efficiencies at retention times of 1.0 s and 2.1 s for the two reactors, *i.e.*, laminated film-electrode and laminated film-electrode packed-bed plasma reactors. High performance is achieved with these reactors because *SE* is relatively low. Furthermore, the performance of the film-electrode packed-bed plasma reactor is better than that of the film-electrode plasma reactor. This may be attributed to the pellets; the ferroelectric pellets between the electrodes cause high ionization around the pellets because of their polarizations. In both the reactors, the *SE* at a retention time of 1.0 s is lower that at 2.1 s. Therefore, the energy efficiency increases, even though the decomposition efficiency decreases at a higher flow rate.

Evaluations of the present plasma reactors and the barrier-type packed-bed plasma reactor [12] are performed by comparing SE values for NH_3_ decomposition. The flow concentration of NH_3_ is unified in each experiment. The SE results to be 4.6, 3.2 and 2.7 (W min/L) for laminated film-electrode, barrier-type packed-bed and laminated film-electrode packed-bed, in descending order. As one of the merits of film-electrode plasma reactors, they can achieve the decomposition at high flow rate.

### 3.5. Chemical Reactions of Odor Removal Using Nonthermal Plasma

The plasma reactors used in this study can generate a nonthermal plasma, in which a highly chemically activated state is realized at a lower input power than that required for a thermal plasma under atmospheric pressure and temperature. The decomposition of an odorous gas using a nonthermal plasma has been reported by the authors [1,3,4,12,15]. The fundamental chemical reactions in the decomposition of NH_3_ using nonthermal plasma are shown below. In the air activated by nonthermal plasma, oxygen radicals (O), ozone (O_3_) and superoxide anion (O_2_^−^) are induced by high-speed electrons (e), and hydroxyl radicals (OH) are generated in the presence of moisture:

(2) $$O_{2}\;+\;e\to 2O$$

(3) $$O_{3}\;\to \;O_{2}\;+\;O$$

(4) $$O_{2}\;+\;e\;\to \;O_{2}^{-}$$

(5) $$O_{2}^{-}\;\to \;O\;+\;O^{-}$$

(6) $$H_{2}O\;+\;O\;\to \;2OH$$

(7) $$O_{3}\;+\;H_{2}O\;+\;h\nu \;\to \;O_{2}\;+\;2OH$$

Odorous gases are decomposed or oxidized, mainly by O and OH. The main chemical reactions between NH_3_ and these radicals are shown below:

(8) $$2NH_{3}\;+\;3O\;\to \;N_{2}\;+\;3H_{2}O$$

(9) $$2NH_{3}\;+\;6OH\;\to \;N_{2}\;+\;6H_{2}O$$

(10) $$2NH_{3}\;+\;5O\;\to \;2NO\;+\;3H_{2}O$$

(11) $$2NH_{3}\;+\;7O\;\to \;2NO_{2}\;+\;3H_{2}O$$

(12) $$2NH_{3}\;+\;4O\;\to \;N_{2}O\;+\;3H_{2}O$$

(13) $$NH_{3}\;+\;4O\;\to \;HNO_{3}\;+\;H_{2}O$$

(14) $$NH_{3}\;+\;HNO_{3}\;\to \;NH_{4}NO_{3}\;(\text{particulate})$$

The decomposition or thermal incineration of NH_3_ begins at temperatures higher than 600 °C. The experiments are carried out at the gas temperature of lower than 240 °C. Thus, the decomposition is due to the plasma. Although reactions (10)–(13) of NO_x_ generation are usually significant in thermal decomposition, they do not progress sufficiently in the nonthermal plasma decomposition [12]. Therefore, in this method, NH_3_ is mainly removed according to reactions (8), (9), (13) and (14), with minimum byproduct generation and high energy efficiency. Byproducts were reported in the literature [3,17]. The byproducts are N_2_O, HNO_3_ and NH_4_NO_3_ as seen in reactions (12)–(14).

## 4. Conclusions

Experiments are carried out to remove NH_3_, the main odor component in living environments, using a laminated film-electrode nonthermal plasma reactor and a laminated film-electrode packed-bed plasma reactor under various conditions; the basic characteristics of the nonthermal reactor are elucidated. The main results can be summarized as follows:

1. NH_3_ removal is investigated by using two types of plasma reactors, a laminated film-electrode plasma reactor and a laminated film-electrode packed-bed plasma reactor. In both plasma reactors, 100% NH_3_ removal can be accomplished using a nonthermal plasma.
2. Specific energy (*SE*) evaluation reveals that the performance of the film-electrode packed-bed plasma reactor is better than that that of the film-electrode plasma reactor.
3. It is possible to decompose NH_3_ using the film-electrode reactor and the film-electrode packed-bed plasma reactor. However, the removal efficiency of the film-electrode reactor is lower than that of the film-electrode packed-bed plasma reactor.
4. NH_3_ removal efficiency is not significantly affected by mixing another odor component such as CH_3_CHO.

## Figures and Tables

**Table 1.** Comparison of specific energy.

**Retention time, s**	**Film-electrode plasma reactor, W min/L**	**Film-electrode packed-bed plasma reactor, W min/L**
1.0	4.2	2.5
2.1	5.0	2.9
